# Supplementary material for: Presynaptic gating of monkey proprioceptive signals for proper motor action
Source: Nat Commun. 2023 Oct 25;14:6537. doi: 10.1038/s41467-023-42077-w (PMC10600222; doi:10.1038/s41467-023-42077-w)
Supplement: Supplementary file 1 — Supplementary Information [file 41467_2023_42077_MOESM1_ESM.pdf]

TITLE

Presynaptic gating of monkey proprioceptive signals for proper motor action

AUTHORS

Saeka Tomatsu[1,2], GeeHee Kim[1,3,4], Shinji Kubota[1], and Kazuhiko Seki[1,3*]

[1]National Center of Neurology and Psychiatry, National Institute of Neuroscience, Tokyo, Japan
[2]Present address: National Institutes of Natural Sciences, National Institute for Physiological Sciences, Aichi, Japan
[3]National Institutes of Natural Sciences, National Institute for Physiological Sciences, Aichi, Japan
[4]Present address: Graduate School of Arts and Sciences, The University of Tokyo, Komaba, Tokyo, Japan

Corresponding author: Kazuhiko Seki (seki@ncnp.go.jp)

SUPPLEMENTARY FIGURES

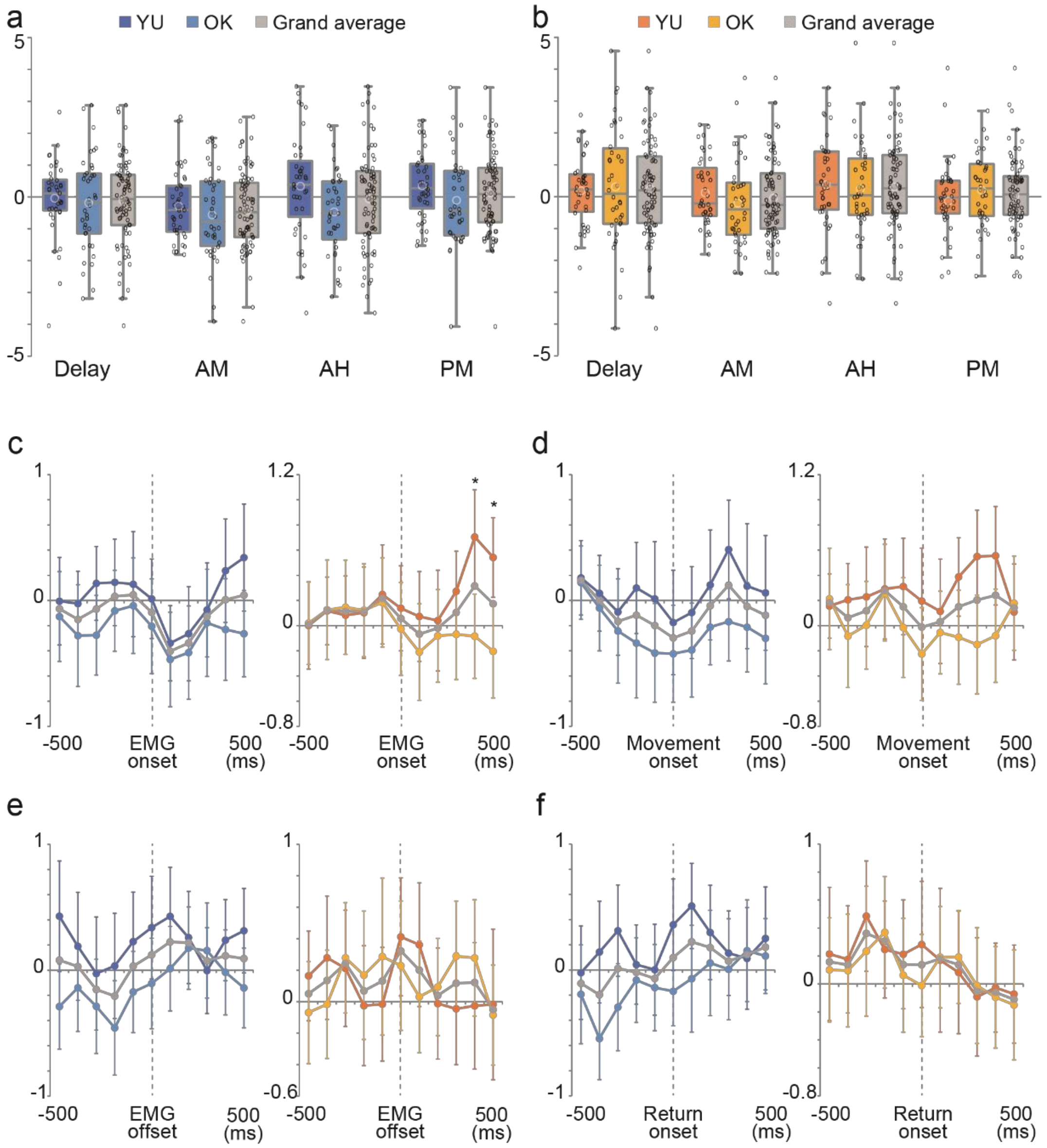

Supplementary Figure 1 | Comparison between two monkeys.

a and **b**. Distribution of antidromic volley (ADV) size in the extension (**a**) and flexion trials (**b**) is presented for each monkey (Monkeys Y, n = 39 ADVs, and Monkey O, n = 38 ADVs) and as the grand average (n = 77 ADVs). AH, Active Hold; AM, Active Movement; PM, Passive Return Movement. B The box plots indicate maximum, minimum, median, upper, and lower quartiles as lines, except for values (dots) exceeding 1.5 times the interquartile range from the upper and lower quartiles. White large circles indicate mean value, and black small circles indicate each data point. **c–f**. Temporal modulation of ADVs for two monkeys with their grand average aligned to electromyography (EMG) onset (c), movement onset (d), EMG offset (e), and movement

offset (f) using the same format as Figure 4. See **a** and **b** for colour coding. Dots indicate average and error bars indicate 95% confidence intervals. *, $p < 0.05$, df = 75, two-tailed t-test comparison of two monkeys using Bonferroni's correction (correction size = 11). The corrected p values at **c** flexion were 0.027 (400 ms) and 0.034 (500 ms).
Monkeys Y, n = 39 ADVs; Monkey O, n = 38 ADVs; and the grand average, n = 77 ADVs.

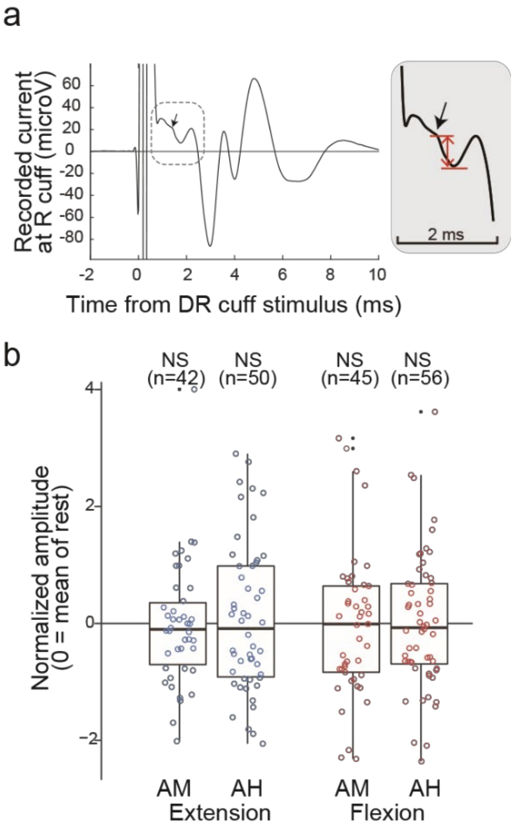

Supplementary Figure 2 | Recording stability of a cuff electrode implanted over the deep radial (DR) nerve during the task.

a. Averaged orthodromic volley evoked in the cuff electrode implanted to the radial (R) nerve. After the artifact of stimulation to the distal cuff, distinct incoming volleys were observed. Average from 857 stimuli. Inset, enlarged trace around the incoming volley (dotted box in the left panel). The peak-to-peak amplitude (red arrow) of the incoming volleys evoked in each behavioural epoch was measured. **b**. Distribution of the normalized amplitude of the volley evoked in each behavioural epoch. Normalization to the Rest epoch was performed for each trial. The box plots indicate maximum, minimum, median, upper, and lower quartiles as lines, except for values (open circles) exceeding 1.5 times the interquartile range from the upper and lower quartiles. Black dots indicate outliers. AH, Active Hold; AM, Active Movement. NS, $p > 0.05$ (extension, AM, $p = 0.869$, AH, $p = 0.902$; flexion, AM, $p = 0.855$, AH, $p = 0.827$), two-sided t-test compared with 0 (Rest).

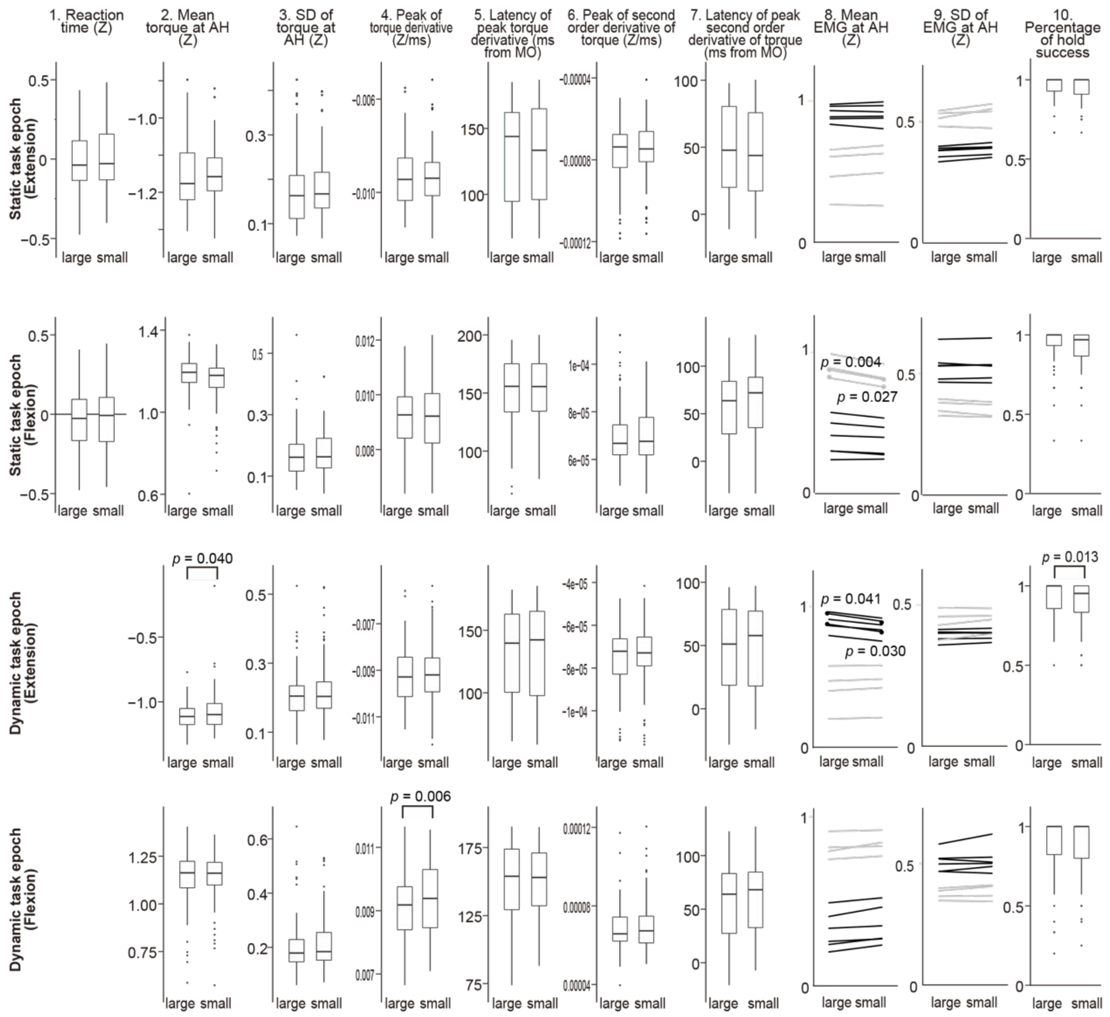

Supplementary Figure 3 | Summary of the comparison between antidromic volley (ADV) modulation and task performance.

Comparison of performance between the large and small ADV trials. Each row represents different task epochs in which the ADV area was measured, and the large and small ADV trial groups were defined. Each column represents 10 different measures of task performance. P values were from two-sided paired t-test (n = 77 ADVs). The box plots indicate maximum, minimum, median, upper, and lower quartiles as lines, except for values (dots) exceeding 1.5 times the interquartile range from the upper and lower quartiles. For the mean and standard deviation (SD) of the electromyography (EMG) traces (#8-9), line plots indicate the mean or SD of the muscle activity recorded from movement onset from +0.3 to +0.7 s (mainly during the Active Hold [AH] epoch). A significant difference between the large and small ADV trials is represented by filled dots (*t*-test, $p < 0.05$). Black lines indicate the results for extensor muscles and grey lines

indicate the results for flexor muscles. Other panels with bar plots illustrate the mean and SD of each metric. Note that reaction times in the dynamic task epochs were not compared.

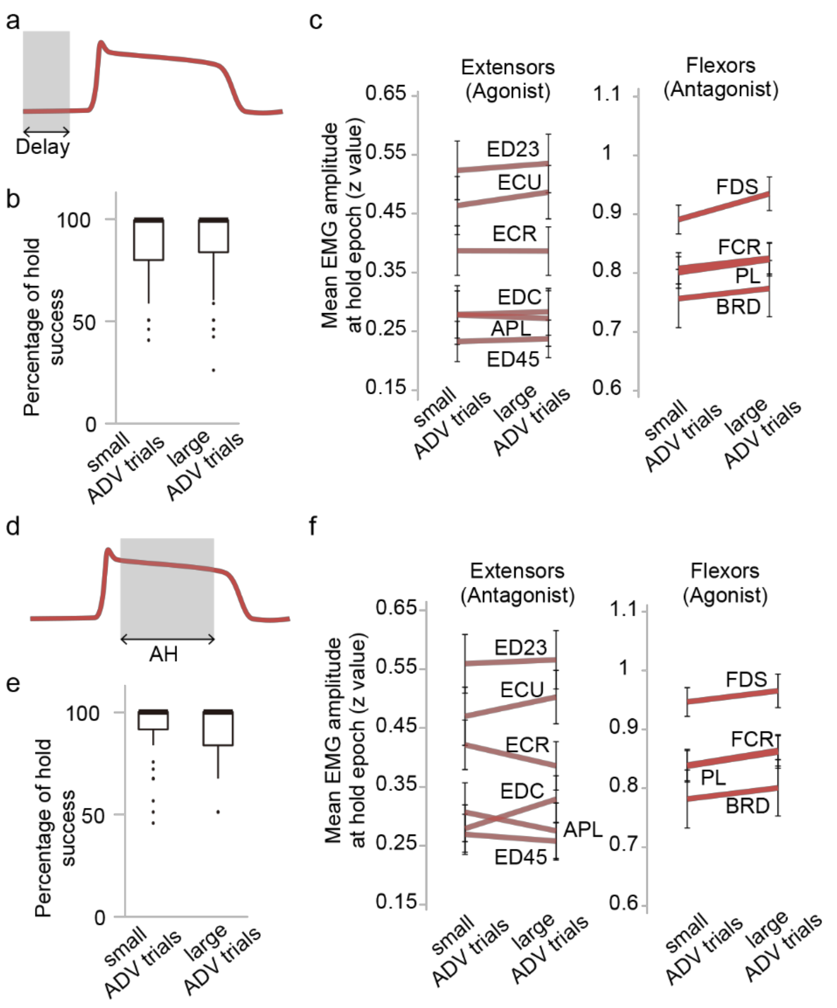

Supplementary Figure 4 | Relationship between antidromic volley modulation at two behavioural epochs in flexion trials and task performance.

a and **d.** Assessment window (shaded area) for computing the mean ADV area for each trial. **b** and **e.** Hold success ratio in the trials classified by the mean ADV in each assessment window. The box plots indicate maximum, minimum, median, upper, and lower quartiles as lines, except for values (dots) exceeding 1.5 times the interquartile range from the upper and lower quartiles. Two-tailed paired t-test indicated not significant difference between two ADV trials in the both cases. **c** and **f.** Means and standard errors of the EMG amplitude of individual wrist extensor and flexor muscles during the Active Hold (AH) epoch of trials classified by the mean ADV in the respective assessment windows. Two-tailed paired t-test indicated not significant difference between two ADV trials in all muscles.

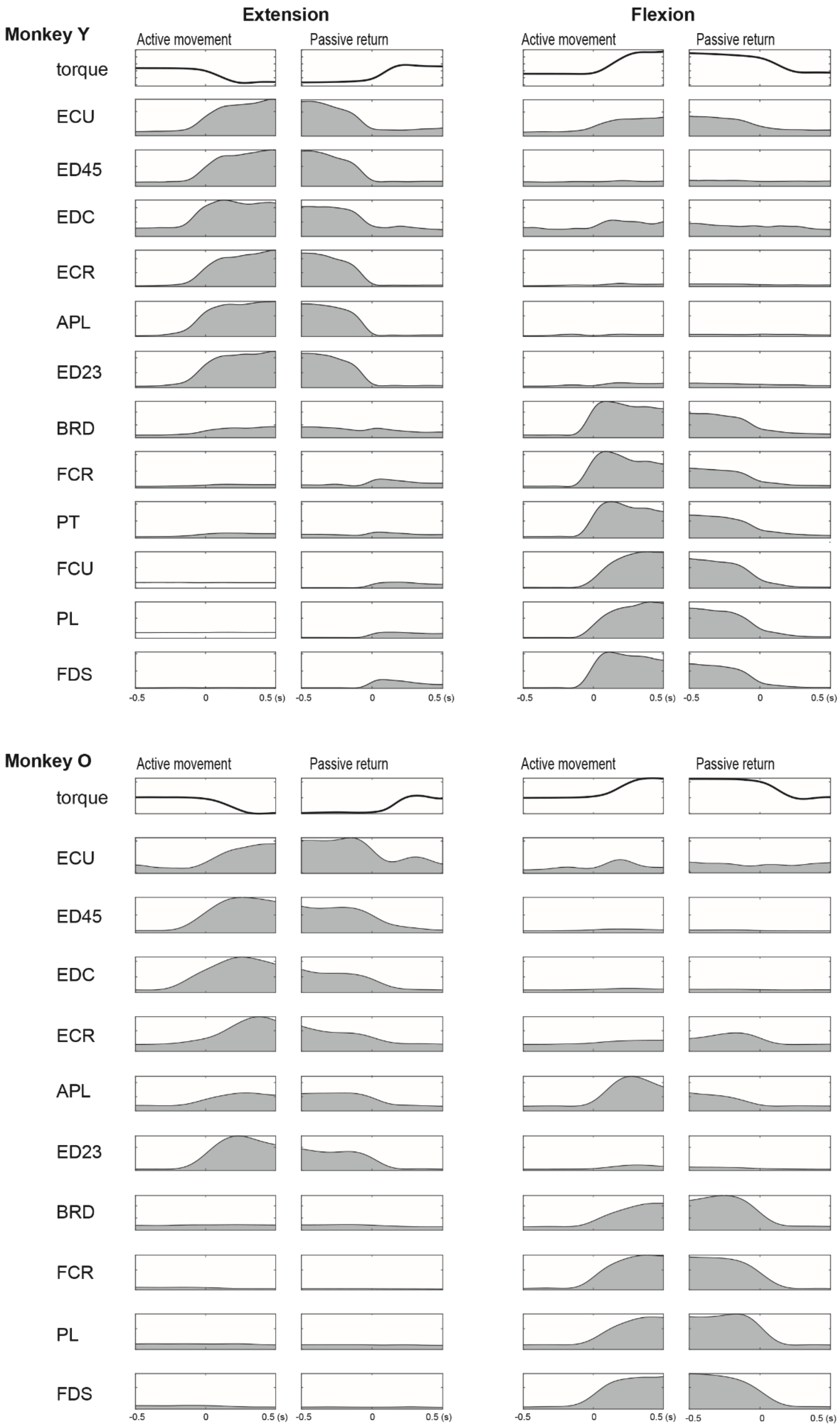
Extension
Flexion
Monkey Y
Active movement
Passive return
Active movement
Passive return
torque
ECU
ED45
EDC
ECR
APL
ED23
BRD
FCR
PT
FCU
PL
FDS
-0.5
0
0.5 (s)
Monkey O
Active movement
Passive return
Active movement
Passive return
torque
ECU
ED45
EDC
ECR
APL
ED23
BRD
FCR
PL
FDS
-0.5
0
0.5 (s)

Supplementary Figure 5 | Example of electromyography (EMG) activity recorded at movement onset or offset in the extension and flexion trials.

Movement onset (left panel in each column) and offset (onset of passive return movement, right panel in each column) are illustrated as 0 on the *x*-axis of each panel (units = s). Data from all perfect trials on a given recording day were averaged and normalized. Recordings of wrist torque are also presented at the top of each panel. The *y*-axis of each panel was normalized with the maximum value of the averaged time series so that the four panels from one muscle are illustrated on the same scale. Note the clear reciprocal activity pattern between flexor and extensor muscles. APL, abductor pollicis longus; BRD, brachioradialis; ECR, extensor carpi radialis; ECU, extensor carpi ulnaris; ED23, extensor digitorum-2,3; ED45, extensor digitorum-4,5; EDC, extensor digitorum communis; FCR, flexor carpi radialis; FCU, flexor carpi ulnaris; FDS, flexor digitorum superficialis; PL, palmaris longus; PT, pronator teres.

ADV #	Monkey	Track #	Spinal segment	Depth from first cell (μm)	Conduction velocity (m/s)	Shape of ADV	Duration of the first wave (ms)	Duration of the second wave (ms)
1	Monkey Y	1	C6	900	69.14	PN	0.11	0.11
2	Monkey Y	2	C6	1360	63.68	NP	0.11	0.18
3	Monkey Y	3	C6	580	71.70	N	0.11	
4	Monkey Y	3	C6	580	69.14	NP	0.11	0.11
5	Monkey Y	4	C6	920	70.14	NP	0.09	0.09
6	Monkey Y	4	C6	920	66.30	NP	0.09	0.14
7	Monkey Y	5	C6	370	72.78	NP	0.14	0.14
8	Monkey Y	5	C6	370	66.76	NP	0.18	0.23
9	Monkey Y	6	C6	1200	62.86	N	0.11	
10	Monkey Y	6	C6	1200	60.12	NP	0.11	0.14
11	Monkey Y	7	C6	—	69.14	NP	0.11	0.23
12	Monkey Y	7	C6	—	51.76	NP	0.18	0.11
13	Monkey Y	8	C6	—	65.85	NP	0.11	0.07
14	Monkey Y	9	C6	10	75.63	NP	0.14	0.20
15	Monkey Y	9	C6	10	54.38	NP	0.16	0.18
16	Monkey Y	10	C5	250	64.53	PN	0.09	0.14
17	Monkey Y	11	C5	710	64.11	PN	0.09	0.11
18	Monkey Y	12	C5	1720	70.66	PN	0.09	0.11
19	Monkey Y	13	C5	3050	59.02	PN	0.14	0.14
20	Monkey Y	14	C5	70	71.18	PN	0.09	0.11
21	Monkey Y	15	C5	940	54.08	NP	0.14	0.09
22	Monkey Y	16	C5	—	61.27	PN	0.14	0.09
23	Monkey Y	16	C5	—	56.28	PN	0.14	0.11
24	Monkey Y	16	C5	—	48.16	NP	0.14	0.14
25	Monkey Y	17	C5	2040	62.45	NP	0.14	0.11
26	Monkey Y	18	C5	—	71.18	PN	0.14	0.11
27	Monkey Y	19	C5	0	59.02	NP	0.11	0.14
28	Monkey Y	20	C6	—	73.33	NP	0.11	0.14
29	Monkey Y	20	C6	—	65.41	PN	0.07	0.09
30	Monkey Y	20	C6	—	62.45	PN	0.07	0.11
31	Monkey Y	20	C6	—	58.67	PN	0.18	0.14
32	Monkey Y	20	C6	—	48.40	NP	0.11	0.14
33	Monkey Y	21	C5	1431	71.18	NP	0.09	0.27
34	Monkey Y	21	C5	1431	63.68	PN	0.18	0.14
35	Monkey Y	21	C5	1431	73.33	NP	0.18	0.23
36	Monkey Y	21	C5	1431	64.53	NP	0.14	0.18
37	Monkey Y	22	C7	880	65.41	NP	0.09	0.14
38	Monkey Y	22	C7	880	61.27	NP	0.14	0.09
39	Monkey Y	22	C7	880	57.62	NP	0.14	0.18
40	Monkey O	23	C8	3004	71.11	PN	0.18	0.20
41	Monkey O	23	C8	3004	59.16	PN	0.25	0.18
42	Monkey O	23	C8	3004	46.62	PN	0.30	0.28
43	Monkey O	24	C8	2	64.59	NP	0.28	0.23
44	Monkey O	24	C8	2	54.57	P	0.25	
45	Monkey O	24	C8	2	47.25	P	0.53	
46	Monkey O	25	C8	1238	67.69	PN	0.15	0.18
47	Monkey O	25	C8	1238	60.17	NP	0.13	0.38
48	Monkey O	25	C8	1238	46.93	NP	0.25	0.15
49	Monkey O	26	C8	307	74.11	PN	0.23	0.18
50	Monkey O	26	C8	307	58.18	NP	0.23	0.15
51	Monkey O	27	C8	571	67.05	PN	0.25	0.20
52	Monkey O	27	C8	571	47.57	N	0.43	
53	Monkey O	28	C8	420	72.58	PN	0.13	0.15
54	Monkey O	28	C8	420	61.75	PN	0.18	0.18
55	Monkey O	28	C8	420	53.33	PN	0.15	0.13
56	Monkey O	29	C8	164	58.67	NP	0.25	0.23
57	Monkey O	29	C8	164	50.65	PN	0.20	0.33
58	Monkey O	29	C8	164	42.93	PN	0.18	0.28
59	Monkey O	30	C8	1335	73.33	NP	0.15	0.18
60	Monkey O	30	C8	1335	64.59	NP	0.30	0.35
61	Monkey O	30	C8	1335	52.15	PN	0.38	0.23
62	Monkey O	30	C8	1335	43.73	PN	0.15	0.15
63	Monkey O	31	C8	1681	60.17	NP	0.18	0.28
64	Monkey O	32	C8	872	76.52	PN	0.13	0.15
65	Monkey O	32	C8	872	68.35	PN	0.13	0.25
66	Monkey O	32	C8	872	59.66	PN	0.18	0.05
67	Monkey O	32	C8	872	55.43	PN	0.15	0.13
68	Monkey O	33	C8	313	79.10	PN	0.15	0.18
69	Monkey O	33	C8	313	69.02	PN	0.18	0.18
70	Monkey O	33	C8	313	55.00	PN	0.25	0.23
71	Monkey O	34	C8	1768	67.05	NP	0.18	0.23
72	Monkey O	35	C8	776	77.36	PN	0.35	0.18
73	Monkey O	35	C8	776	62.86	PN	0.18	0.20
74	Monkey O	35	C8	776	55.43	PN	0.13	0.08
75	Monkey O	36	C7	370	62.86	PN	0.43	0.28
76	Monkey O	36	C7	776	50.29	PN	0.38	0.15
77	Monkey O	36	C7	776	43.73	PN	0.18	0.23

Supplementary Table 1 | Fundamental characteristics of individual antidromic volleys (ADVs).

The conduction velocity of ADVs was calculated using the ADV onset latency and the distance between the distal electrode in the nerve cuff (a platinum plate on the proximal side) and the spinal cord entry point of the dorsal root at segment C6. The distance was measured post-mortem by placing a wet silk thread between the exposed radial nerve and the most prominent stream of the spinal dorsal root. The depth of the intraspinal site is expressed as the distance relative to the intraspinal depth where the first distinct neural activity was observed, most likely from a dorsal horn neuron in each electrode penetration. Thus, when no significant neural activity was detected before reaching the intraspinal site where ADVs were evoked, their intraspinal depth could not be represented (noted as '-'). N, negative peak; NP, negative–positive peak; P, positive peak; PN, positive–negative peak.

	Success	Error		
(%)		Short hold	Opposite	No movement
Extension	74.46	14.75	4.53	6.26
Flexion	54.58	13.13	25.68	6.61

Supplementary Table 2 | Distribution of successful trials and three different types of error trials.

Success: a trial with wrist torque in the correct movement direction and sufficient sustained duration. Short hold: a trial with wrist torque in the correct movement direction, but without sufficient sustained duration. Opposite: a trial with wrist torque in the wrong movement direction. No movement: a trial with no change in wrist torque for 0.5 s after the Go cue. Data from all trials performed by two monkeys (n = 6,219) while recording ADVs (from 36 intraspinal sites) were used for computing each percentage and grand averaged.

SUPPLEMENTARY NOTE 1

Measurement of PSI modulation by the size of ADVs evoked in muscle afferents.

Here, we briefly explain how the ADV size evoked in DR afferents could reflect PSI modulation during movement. The activation of $GABA_A$ receptors at afferent terminals triggers the efflux of Cl^- because the intracellular Cl^- concentration in primary afferents is higher than in the extracellular space[1]. This efflux depolarizes the terminal, which we record as PAD. During PAD, the shunting effect and the inactivation of voltage-activated cation channels interrupt the intracellular increase of Ca^{2+} and transmitter release. As a consequence, the level of PAD positively reflects $GABA_A$-mediated PSI (but[2], see Discussion). Therefore, we assessed the task-dependent modulation of PAD at muscle afferent terminals.

In experiments using immobilized animals under anaesthesia, PAD modulation has been evaluated frequently by intraspinal threshold changes to repetitive microstimulation in intra- and extra-axonal recordings of sectioned dorsal root[3]. The all-or-non threshold of single afferent potentials was measured by applying repetitive intraspinal pulses. Since this method cannot be applied to awake, behaving monkeys, we established a new method[4 5] based on Wall's technique[6] that measures the ADVs of the populations of afferent fibres recorded in whole afferent nerves elicited by intraspinal microstimulation. Similar to the earlier work that applied Wall's technique during locomotion[7] and voluntary movements in cats[8], we assumed that larger ADVs represent larger PAD.

Amplitude changes could reflect either changes in the firing probability of a single axon or the variable recruitment of multiple axons with nearly identical conduction velocities, or both[5]. For a detailed discussion about the relationship between the shape of ADVs and PSI, readers could refer to our previous report[5]. In this paper, we elicited and analysed ADVs using the same method as in our previous reports[4, 5], by including one additional restriction to meet the mixed-nerve nature of the DR nerve (see the **Dissociating antidromic and orthodromic volleys** section in the **METHODS**).

SUPPLEMENTARY NOTE 2

Influence of other factors on the waveforms of ADVs.

One might argue that the modulation of ADV size as shown in Fig. 2 might represent an unwanted consequence of other non-physiological factors. However, we concluded, similar as in our previous report on the ADVs of cutaneous afferents[5], it is less likely for the following reasons.

First, cross-talk from the activity of muscles surrounding the nerve cuffs is unlikely because we already confirmed, using almost the same experimental setup as in the present study, that the signal recorded in the nerve cuff is independent of the activity of the surrounding muscles (see Fig. 2 in Seki et al. 2009[5]).

Second, to exclude potential contamination from orthodromic volleys, which might be evoked in DR motor axons, we carefully adjusted the stimulus current while monitoring EMG in the wrist extensor muscles (see the **Dissociating antidromic and orthodromic volleys** section in the **METHODS**).

Third, a subtle mechanical movement and stress applied during wrist movement might change the recording efficacy of the nerve cuff electrode. We excluded this possibility by performing an additional experiment. As shown in Supplementary Fig. 2, we found no difference in the size of orthodromic volleys recorded by DR cuff electrode during the AM and AH periods in the flexion and extension trials. Therefore, together with the comparable confirmation in our previous report on the SR nerve[9], we concluded that recording efficacy was constant throughout the different task epochs.

Fourth, theoretically speaking, the size of ADV waveforms could be altered if they collide with reafferent orthodromic spikes in the same axons. Although making a realistic assumption of such collisions occurring is challenging because the necessary information has not been reported yet, it would be worthwhile estimating this using available pieces of information. Here, we assume that all Ia and Ib fibres in the DR afferents were recruited during the task. First, the conduction time of the DR nerve from the cuff to the cord was approximately 2.7 ms (2.6 ms for Monkey O, and 2.8 ms for Monkey Y; see also Fig. 1 in our previous report[10]). Consequently, the probability that any afferent spikes being conducted orthodromically between the cuff to the cord, that is the chance of a given ADV colliding with them, can be obtained as follows:

$$\text{Collision probability} = 2.7\ \text{ms} / \text{mean inter-spike interval (i.s.i., ms)} \qquad (1)$$

The firing rate of the dorsal root ganglion afferents (unidentified) involved in the spinal reflex in monkeys performing a comparable wrist movement can be estimated as 10–40 Hz (25–100 ms i.s.i.) [11]. By fitting this estimation into equation 1, we obtain 2.7% (minimal) to 10.8% (maximal) as the probability of a collision. Note that this is likely to be an overestimation because the type-identified human wrist extensor Ia afferents fire at less than 10 Hz[12]. Moreover, the assumption of the recruitment of all Ia and Ib fibres could represent an overestimation of weaker muscle contractions[13] as in this study. Anyway, by assuming a 2–10% reduction of ADV size might be associated with a collision, then the modulation of 4% (n = 2/77 showing a <2% reduction) to 18% (n = 14/77 showing a <10% reduction) of ADVs might be explained by collisions. Therefore, we can conclude that the influence of collisions on ADV modulation is minor.

SUPPLEMENTARY REFERENCES

1. Alvarez-Leefmans FJ, Gamino SM, Giraldez F, Nogueron I. Intracellular chloride regulation in amphibian dorsal root ganglion neurones studied with ion-selective microelectrodes. *J Physiol* **406**, 225-246, doi: 10.1113/jphysiol.1988.sp017378 (1988).
2. Hari K*, et al.* GABA facilitates spike propagation through branch points of sensory axons in the spinal cord. *Nat Neurosci*, **25**, 1288-1299, doi: 10.1038/s41593-022-01162-x (2022).
3. Cote MP, Gossard JP. Task-dependent presynaptic inhibition. *J Neurosci* **23**, 1886-1893, doi: 10.1523/JNEUROSCI.23-05-01886.2003 (2003).
4. Seki K, Perlmutter SI, Fetz EE. Sensory input to primate spinal cord is presynaptically inhibited during voluntary movement. *Nat Neurosci* **6**, 1309-1316, doi: 10.1038/nn1154 (2003).
5. Seki K, Perlmutter SI, Fetz EE. Task-dependent modulation of primary afferent depolarization in cervical spinal cord of monkeys performing an instructed delay task. *J Neurophysiol* **102**, 85-99, doi: 10.1152/jn.91113.2008 (2009).
6. Wall PD. Excitability changes in afferent fibre terminations and their relation to slow potential. *Journal of Physiology* **142**, 1-21 (1958).
7. Duenas SH, Rudomin P. Excitability changes of ankle extensor group Ia and Ib fibers during fictive locomotion in the cat. *Exp Brain Res* **70**, 15-25, doi: 10.1007/BF00271842 (1988).
8. Ghez C, Pisa M. Inhibition of afferent transmission in cuneate nucleus during voluntary movement in the cat. *Brain Res* **40**, 145-155, doi: 10.1016/0006-8993(72)90120-5 (1972).
9. Seki K, Fetz EE. Gating of Sensory Input at Spinal and Cortical Levels during Preparation and Execution of Voluntary Movement. *J Neurosci* **32**, 890-902, doi: 10.1523/JNEUROSCI.4958-11.2012 (2012).
10. Confais J, Kim G, Tomatsu S, Takei T, Seki K. Nerve-Specific Input Modulation to Spinal Neurons during a Motor Task in the Monkey. *J Neurosci* **37**, 2612-2626, doi: 10.1523/JNEUROSCI.2561-16.2017 (2017).
11. Flament D, Fortier PA, Fetz EE. Response patterns and postspike effects of peripheral afferents in dorsal root ganglia of behaving monkeys. *J Neurophysiol* **67**, 875-889, doi: 10.1152/jn.1992.67.4.875 (1992).
12. Jones KE, Wessberg J, Vallbo AB. Directional tuning of human forearm muscle afferents during voluntary wrist movements. *J Physiol* **536**, 635-647, doi: 10.1111/j.1469-7793.2001.0635c.xd (2001).

13. Edin BB, Vallbo AB. Muscle afferent responses to isometric contractions and relaxations in humans. *J Neurophysiol* **63**, 1307-1313, doi: 10.1152/jn.1990.63.6.1307 (1990).
