# Supplementary material for: Presynaptic gating of monkey proprioceptive signals for proper motor action
Source: Nat Commun. 2023 Oct 25;14:6537. doi: 10.1038/s41467-023-42077-w (PMC10600222; doi:10.1038/s41467-023-42077-w)
Supplement: Supplementary file 2 — Reporting Summary [file 41467_2023_42077_MOESM2_ESM.pdf]

Corresponding author(s): Kazuhiko SEKI

Last updated by author(s): Jul 23, 2023

Reporting Summary

Nature Portfolio wishes to improve the reproducibility of the work that we publish. This form provides structure for consistency and transparency in reporting. For further information on Nature Portfolio policies, see our Editorial Policies and the Editorial Policy Checklist.

Statistics

For all statistical analyses, confirm that the following items are present in the figure legend, table legend, main text, or Methods section.

n/a	Confirmed	
☐	☒	The exact sample size (*n*) for each experimental group/condition, given as a discrete number and unit of measurement
☐	☒	A statement on whether measurements were taken from distinct samples or whether the same sample was measured repeatedly
☐	☒	The statistical test(s) used AND whether they are one- or two-sided<br>*Only common tests should be described solely by name; describe more complex techniques in the Methods section.*
☐	☒	A description of all covariates tested
☐	☒	A description of any assumptions or corrections, such as tests of normality and adjustment for multiple comparisons
☐	☒	A full description of the statistical parameters including central tendency (e.g. means) or other basic estimates (e.g. regression coefficient) AND variation (e.g. standard deviation) or associated estimates of uncertainty (e.g. confidence intervals)
☐	☒	For null hypothesis testing, the test statistic (e.g. *F*, *t*, *r*) with confidence intervals, effect sizes, degrees of freedom and *P* value noted<br>*Give P values as exact values whenever suitable.*
☒	☐	For Bayesian analysis, information on the choice of priors and Markov chain Monte Carlo settings
☒	☐	For hierarchical and complex designs, identification of the appropriate level for tests and full reporting of outcomes
☒	☐	Estimates of effect sizes (e.g. Cohen's *d*, Pearson's *r*), indicating how they were calculated

Our web collection on statistics for biologists contains articles on many of the points above.

Software and code

Policy information about availability of computer code

Data collection	Data were amplified and filtered by MCP-Plus (AlphaOmega, Israel) and digitized by DAP4200a/526 (Microstar Laboratories, USA) for a monkey, or amplified, filtered, and digitized by AlphaLabSnR (AlphaOmega, Israel) for another monkey. The experiments were controlled by special-purposed software (TEMPO; Reflective Computing, Olympia, WA, USA).
Data analysis	Statistical and machine learning toolbox (ver. 12.3), signal processing toolbox (ver. 9.0), parallel computing toolboox (ver. 7.6), control system toolboox (ver. 10.11.1) provided by Matlab R2020b (Mathworks Inc., Natick, MA, U.S.A.). Fast ICA package (ver. 1.2-3), EZR package (ver. 1.60), ggplot2 package (ver. 3.3.6), ggdist package (ver. 3.2.0) provided by R (ver. 4.2.0) and RStudio (ver. 2022.12.0+353).

For manuscripts utilizing custom algorithms or software that are central to the research but not yet described in published literature, software must be made available to editors and reviewers. We strongly encourage code deposition in a community repository (e.g. GitHub). See the Nature Portfolio guidelines for submitting code & software for further information.

Data

Policy information about availability of data

All manuscripts must include a data availability statement. This statement should provide the following information, where applicable:

- Accession codes, unique identifiers, or web links for publicly available datasets
- A description of any restrictions on data availability
- For clinical datasets or third party data, please ensure that the statement adheres to our policy

The datasets generated during and/or analysed during the current study are available from https://github.com/saetoma/Tomatsu_NC2023.git.

Human research participants

Policy information about studies involving human research participants and Sex and Gender in Research.

Reporting on sex and gender	N/A
Population characteristics	N/A
Recruitment	N/A
Ethics oversight	N/A

Note that full information on the approval of the study protocol must also be provided in the manuscript.

Field-specific reporting

Please select the one below that is the best fit for your research. If you are not sure, read the appropriate sections before making your selection.

☒ Life sciences ☐ Behavioural & social sciences ☐ Ecological, evolutionary & environmental sciences

For a reference copy of the document with all sections, see nature.com/documents/nr-reporting-summary-flat.pdf

Life sciences study design

All studies must disclose on these points even when the disclosure is negative.

Sample size	We recorded 77 antidromic peripheral nerve volleys elicited by microelectrical stimulation at 37 intraspinal sites. The 36 sites were from 2 monkeys (39 volleys in 23 sites from a male and 38 volleys in 14 sites from a female). In accordance with 3R principle, we used the minimal number of animals, and repeated the experiment for each animal with different intraspinal sites.
Data exclusions	Because of its anatomical restriction, our data was easily to be contaminated by responses derived from muscle nerves. To prevent it, we excluded 4 data which indicated muscle twitch by intraspinal microstimulation.
Replication	Two monkeys generally exhibited the common results, indicating replication of our data.
Randomization	We used wrist movement for two opposite directions as the experimental condition, and randomized the order to make the animals not to predict the next direction until a directional cue was appeared.
Blinding	We were blinded to group allocation during data collection.

Reporting for specific materials, systems and methods

We require information from authors about some types of materials, experimental systems and methods used in many studies. Here, indicate whether each material, system or method listed is relevant to your study. If you are not sure if a list item applies to your research, read the appropriate section before selecting a response.

Materials & experimental systems

n/a	Involved in the study
☒	☐ Antibodies
☒	☐ Eukaryotic cell lines
☒	☐ Palaeontology and archaeology
☐	☒ Animals and other organisms
☒	☐ Clinical data
☒	☐ Dual use research of concern

Methods

n/a	Involved in the study
☒	☐ ChIP-seq
☒	☐ Flow cytometry
☒	☐ MRI-based neuroimaging

Animals and other research organisms

Policy information about studies involving animals; ARRIVE guidelines recommended for reporting animal research, and Sex and Gender in Research

Laboratory animals	Japanese monkey , macaca fuscata, 7 years-old
Wild animals	This study did not involve the wild animal.
Reporting on sex	Our animals are one male and one female. So our data did not have a bias originated by sex difference.
Field-collected samples	The study did not involve sample collected from the field.
Ethics oversight	Our experiments were approved by the Institutional Animal Care and Use Committees at the National Institute for Physiological Sciences (NIPS), Aichi, Japan and the National Center of Neurology and Psychiatry (NCNP), Tokyo, Japan.

Note that full information on the approval of the study protocol must also be provided in the manuscript.
